# Risk Factors for Nonsyndromic Orofacial Clefts Among Saudi Children

**DOI:** 10.1002/cre2.70108

**Published:** 2025-03-07

**Authors:** Najla S. Alrejaye, Mostafa A. Abolfotouh, Fathima Fazrina Farook, Elaf Mubarak Abdullah Algharbi, Abdulmajeed Mohammed B. Alharbi, Halah Ibrahim Alshuaibi, Mai Saad Bin Akresh, AlAnood Naif Bin Saedan, Nouf Besher Albesher, Atheer Sami Aldaham, Lujain Ahmad Alghrairy, Latifa Yousef AlGudaibi, Rana Abdullah Alolaiq, Afnan Turki Alzomaili, Mosleh S. Alharbi

**Affiliations:** ^1^ Department of Dental Services Division of Orthodontics, King Abdulaziz Medical City, Ministry of National Guard Health Affairs Riyadh Kingdom of Saudi Arabia; ^2^ Department of Preventive Dental Science College of Dentistry King Saud bin Abdulaziz University for Health Sciences Riyadh Kingdom of Saudi Arabia; ^3^ King Abdullah International Medical Research Center, Ministry of National Guard Health Affairs Riyadh Kingdom of Saudi Arabia; ^4^ King Abdullah International Medical Research Center, King Saud bin Abdulaziz University for Health Sciences Ministry of National Guard Health Affairs Riyadh Kingdom of Saudi Arabia; ^5^ Family Health Department High Institute of Public Health, Alexandria University Alexandria Egypt; ^6^ College of Dentistry King Saud bin Abdulaziz University for Health Sciences Riyadh Kingdom of Saudi Arabia; ^7^ Private Sector Riyadh Kingdom of Saudi Arabia; ^8^ King Saud Medical City Riyadh Kingdom of Saudi Arabia; ^9^ Department of Dental Services King Abdulaziz Medical City, Ministry of National Guard Health Affairs Riyadh Kingdom of Saudi Arabia

**Keywords:** cleft lip and/or palate, environmental factors, etiology, genetic factors

## Abstract

**Objectives:**

The aim of this study was to identify the risk factors associated with nonsyndromic orofacial clefts (NSOFCs) among Saudi children.

**Materials and Methods:**

A case–control study was carried out at the Ministry of National Guard Health Affairs. Cases were children with NSOFCs who were matched by gender and year of birth to healthy controls from the same setting. Data on risk factors were collected by interviewing parents of both cases and controls using a validated questionnaire. The questionnaire consisted of the father's and mother's information and the child's information. The level of significance was set at 0.05. Odds ratio (OR) and 95% confidence intervals (CIs) were used to determine the associated risk factors with NSOFCs.

**Results:**

A total of 188 children were included (88 cases and 100 controls), with a mean age of 5.1 ± 2.3 years. Maternal fever during pregnancy was associated with a significantly higher risk of NSOFCs (OR = 3.4, 95% CI: 0.05–2.5, *p* < 0.05). Additionally, the presence of maternal relatives with orofacial clefts increased the risk (OR = 6.02, 95% CI: 0.43–3.16, *p* < 0.001), whereas the strongest predictor was paternal relatives with orofacial clefts (OR = 8.00, 95% CI: 0.41–3.75, *p* = 0.014). These findings are of utmost importance for the understanding and potential prevention of NSOFCs.

**Conclusions:**

The presence of paternal or maternal relatives with orofacial clefts and maternal fever during the first trimester were predictors for NSOFCs, with having affected paternal relatives being the strongest predictor.

## 1 Introduction

Nonsyndromic orofacial cleft (NSOFC) is the most common craniofacial anomaly, which results from a failure in growth or fusion of the craniofacial developmental processes (Kawalec et al. 2015). However, the exact etiology behind this is complex or largely unknown, as it involves the interaction of multiple genetic and environmental risk factors. The etiology for NSOFC has been extensively studied, and numerous risk factors have been revealed throughout the years, including family history, consanguinity, tobacco smoking, alcohol consumption, malnutrition, infections, drugs, and teratogens (Kawalec et al. 2015).

The genetic aspect of NSOFC etiology has been clearly shown in segregation analysis and twin studies (Marazita et al. 1984; Melnick 2003). Sivertsen et al. (2008) showed that the relative risk of recurrence among first‐degree relatives in a Norwegian population was 32 for cleft lip with or without cleft palate (CP) and 56 for cleft palate only (CPO). Jamilian et al. (2017) conducted a case–control study on syndromic and non‐syndromic Iranian children with cleft lip and/or palate (CL/P). They reported that a family history of clefts was strongly associated with an increased risk of CL/P, particularly in consanguineous marriages.

OFC has been associated with maternal risk factors, including tobacco smoking, corticosteroid use, folic acid deficiency, zinc deficiency, and maternal grief. Burg et al. (2016) reviewed CPO and demonstrated that CPO has a strong genetic component based on its high recurrence rate. They also reported that maternal smoking was the strongest risk factor for CPO. Sabbagh et al. (2016) conducted a case–control study in the western region of Saudi Arabia and found a significantly higher risk of NSOFC with maternal common cold/flu during the 3‐month pregestational period, antiemetic medication, maternal severe morning sickness, maternal use of antibiotics in the first trimester, paternal water‐pipe smoking, maternal passive smoking, and twin births.

NSOFC has presented ethnic and geographic variations that may influence its etiology and prevalence worldwide (Mossey et al. 2009). More studies are needed in the Middle East in general and in Saudi Arabia in particular to further explore the etiology of NSOFC. Therefore, this study aimed to investigate the various potential risk factors and identify those associated with NSOFC among Saudi children.

## 2 Materials and Methods

This retrospective case–control study evaluated the correlation between oral clefts and possible risk factors. It was carried out in the Ministry of National Guard Health Affairs (MNGHA), Riyadh, Kingdom of Saudi Arabia (KSA), from 2022 to 2023. MNGHA receives craniofacial cases from multiple regions and has a tertiary medical city, a children's hospital, a health science university, and a medical research center.

All patients diagnosed with NSOFC, born between January 2014 and March 2023, and treated at MNGHA were included in the study. The exclusion criteria were individuals with multiple congenital anomalies or diagnosed syndromes. Control healthy children (without congenital anomalies) were matched based on gender and age (± 6 months' difference). Before starting data collection, an ethical approval was obtained from the Institutional Review Board of the MNGHA (Ref#. RC20/148/R and RSS22R/015/07).

The sample size for this study was calculated using OpenEpi (Version 3) based on a pilot sample from our data, where 30% of cases (children with NSOFCs) and 7% of controls (children without NSOFCs) had a family history of orofacial clefts (OFCs). With a power of 90% and a confidence level of 95%, the estimated sample size was 143 subjects. A total of 188 subjects (88 cases and 100 controls) were included in the study to ensure that the study is sufficiently powered to detect a significant association between OFC and NSOFC family history.

The treating dentists conducted recruitment and data collection between August 2022 and July 2023 using a structured interview questionnaire validated by experts' opinions, ensuring its content validity. A pilot study assessed its reliability, achieving a Cronbach's alpha of 0.82, confirming internal consistency. The information was collected in a data collection form through an interview with the patient's parents after obtaining their voluntary participation via informed written consent. The study was conducted in accordance with the Declaration of Helsinki (2002 version, available at: http://www.wma.net/e/policy/b3.htm). The participants' personal information was anonymously treated to ensure their privacy and confidentiality, and their data were kept secure. The data collection form was filled by the researchers on the spot using an English questionnaire. The questionnaire was composed of three sections: father's, mother's, and child's information, with a total of 62 questions. An expert in craniofacial orthodontics examined all participants to verify the cleft type.

### 2.1 Statistical Analysis

Categorical variables were compared between the case and control groups using the Chi‐square test or Fisher's exact test, as appropriate. Continuous variables were analyzed using the *t*‐test. Demographic characteristics of the children, mothers, and fathers, along with variables related to genetic factors and parental medical history, were analyzed separately. Unadjusted (uOR) and adjusted odds ratios (aOR), with their 95% confidence intervals (CIs), were calculated to evaluate the associations between various factors and the presence of NSOFC. Firth logistic regression was used to address potential issues related to separation and small sample sizes. All statistical tests were considered significant at a level of 0.05 or less. Analyses were performed using NCSS statistical software (version 2020, NCSS LLC, Kaysville, UT, USA).

## 3 Results

Individuals with other congenital anomalies, such as cardiac defects or missing kidneys, were excluded. A total of 88 verified cases were identified (mean age 5.05 + 2.33 years), along with 100 controls (mean age 5.18 + 2.33 years). Table 1 shows the cleft‐type distribution according to gender for the included cases. The analysis results comparing cases with NSOFC and controls are presented in Table 2. The data were categorized based on various factors, shedding light on potential influences on the occurrence of NSOFC. Notably, the gender distribution among cases and controls had a balanced representation, with no significant difference observed (*p* = 0.935).

**Table 1:** Cleft‐type distribution according to gender, *n* (%).

	Cleft classification			
Gender	CL	CLP	CP	Total
Male	16 (18.18)	26 (29.55)	5 (5.68)	47 (53.41)
Female	15 (17.05)	9 (10.23)	17 (19.32)	41 (46.59)
Total	31 (35.23)	35 (39.77)	22 (25.00)	88 (100)

Abbreviations: CL, cleft lip with or without alveolus; CLP, cleft lip and palate; CP, cleft palate.

**Table 2:** Comparison between the cases and controls on sociodemographic characteristics.

Demographics	Cases *n* (%) 88 (46.81)	Control *n* (%) 100 (53.19)	*p* value
*Child*			
Gender			
Male	47 (53.4)	54 (54)	0.935
Female	41 (23.86)	46 (26)	
Birth weight (kg)			
< 2.5	13 (14.77)	14 (14)	0.273
2.5–3.99	61 (69.32)	59 (59)	
≥ 4	1 (1.14)	4 (4)	
Do not know	13 (14.77)	23 (23)	
*Mother*			
Maternal age (years)			
18–35	67 (76.14)	74 (74)	0.434
> 35	21 (23.86)	26 (26)	
Education level			
Primary school	14 (43.75)	18 (56.25)	0.922
High school	29 (46.77)	33 (53.23)	
Higher education	45 (47.87)	49 (52.13)	
Employment status			
Unemployed	65 (74.71)	77 (77)	0.744
Non‐health sector employee	16 (18.39)	17 (17)	
Health sector employee	6 (6.9)	5 (5)	
Retired	0	1 (1)	
Residence			
Urban	56 (63.64)	68 (68)	0.317
Rural	32 (36.36)	32 (32)	
Marital status during pregnancy			
Living with the father of the child	88 (100)	99 (99)	0.532
Divorced	0	1 (1)	
Widowed	0	0	
*Father*			
Age of the father at child‐birth (years)			
18–35	41 (47.13)	53 (59.6)	0.182
36–50	45 (51.72)	38 (38.38)	
51–60	1 (1.15)	2 (2.02)	
> 60	0	0	
Education level			
Primary	9 (33.33)	18 (66.67)	0.68
High school	38 (45.24)	46 (54.76)	
Higher education	41 (53.25)	36 (46.75)	
Employment			
Unemployed	0	3 (3)	0.289
Non‐health sector employee	69 (78.4)	76 (76)	
Health sector employee	13 (14.77)	11 (11)	
Retired	6 (6.82)	10 (10)	
Family income			
≤ 15,000	55 (51.75)	59 (48.25)	0.103
> 15,000	27 (37.21)	16 (62.79)	

*Note: p* values for differences based on the chi‐squared test for proportions and Fisher's exact test.

Birth weight categories (< 2.5, 2.5–3.99, and ≥ 4 kg) did not show a statistically significant difference between cases and controls (*p* = 0.273). Similarly, the age of both parents at the child's birth, education level of both parents, employment status of both parents, and family income showed no significant differences between cases and controls. None of the mothers were under 18 years of age at the child's birth. Additionally, the mother's residence and marital status during pregnancy did not display significant differences between cases and controls (Table 2). Interestingly, the smoking habits of the father during cleft pregnancy, including whether he smoked inside the house and the average number of smoked cigarettes per day, did not show significant differences between cases and controls (*p* = 0.355, *p* = 0.795, *p* = 0.551, respectively) (Table 3).

**Table 3:** Effect of paternal and maternal factors on NSOFC: Analysis of medical history, smoking, conception methods, and other maternal variables.

Factors	Cases *n* (%) 88 (46.81)	Control *n* (%) 100 (53.19)	*p* value
Presence of any paternal medical condition			
Yes	19 (45.24)	23 (54.76)	0.817
No	69 (47.26)	77 (52.74)	
Paternal smoking during the pregnancy with the studied child			
Yes	35 (40.7)	47 (47.47)	0.355
No	51 (59.3)	52 (52.53)	
Paternal smoking inside the house			
Yes	10 (11.5)	13 (13.1)	0.795
No	27 (31)	34 (34.3)	
Paternal average amount of smoked cigarettes/day			
Nonsmoker	55 (62.5)	51 (52)	0.551
≤ 10	14 (15.9)	19 (19.4)	
11–19	12 (13.6)	18 (18.4)	
≥ 20	7 (8)	10 (10.2)	
Presence of any maternal medical condition			
Yes	71 (80.68)	83 (83)	0.680
No	17 (19.32)	17 (17)	
Employment of conception‐enhancing methods			
Yes	14 (16.28)	5 (5.05)	0.012*
No	72 (83.72)	94 (94.95)	
Maternal use of conception‐enhancing method			
None	72 (88.89)	94 (95.92)	0.171
Fertility medications	5 (6.17)	1 (1.02)	
In vitro fertilization	3 (3.7)	3 (3.06)	
Other	1 (1.23)	0	
Miscarriages before being pregnant with the studied child's			
Yes	32 (37.21)	27 (27.55)	0.161
No	54 (62.79)	71 (12.45)	
Maternal consumption of dietary supplements			
Yes	71 (88.75)	83 (86.46)	0.647
No	9 (11.25)	13 (13.54)	
Maternal consumption of caffeinated drinks			
Yes	79 (46.75)	90 (53.25)	0.959
No	9 (47.37)	10 (52.63))	
Coffee (cups per day)			
None	8 (44.44)	10 (55.56)	0.038*
0 to < 1/month	0	0	
1/month	5 (55.56)	4 (44.44)	
1/day	27 (34.62)	51 (65.38)	
2/day	33 (62.26)	20 (37.74)	
3/day	14 (50)	14 (50)	
Maternal experience with second‐hand smoke exposure during pregnancy			
Yes	16 (19.51)	21 (22.11)	0.672
No	66 (80.49)	74 (77.89)	
Maternal use of pregnancy luteal support medications			
Yes	33 (53.23)	29 (46.77)	0.216
No	55 (43.65)	71 (56.35)	
Maternal use of any other medications during pregnancy			
Yes	45 (60)	30 (40)	0.021*
No	40 (42.11)	55 (57.89)	
Presence of any of the following maternal conditions during the first trimester?			
None	61 (43.26)	80 (56.74)	0.025*
Fever	14 (77.78)	4 (22.22)	
Bleeding	5 (71.43)	2 (28.57)	
Hypertension	0	4 (100)	
Convulsions	0	1 (100)	
Diabetes	3 (37.5)	5 (62.5)	
X‐ray exposure	2 (100)	0	
Other	3 (42.86)	4 (57.14)	
Maternal stress or anxiety during pregnancy			
Yes	36 (54.55)	30 (45.45)	0.54
No	48 (41.03)	69 (58.97)	

* Differences significant at *p* ≤ 0.05.

The familial characteristics of the study population were analyzed to discern potential associations with the occurrence of NSOFC (Table 4). The presence of OFC in the father showed a statistically significant association with NSOFC occurrence in the child (*p* = 0.014*). Cases showed a higher prevalence of OFC among fathers (5.88%) compared to controls, where it was absent (0) (Table 4). Further exploration of familial associations indicated that cases were more likely to have fathers with relatives with OFC compared to controls (*p* = 0.001*). The degree of relationship with affected relatives varied, with no statistically significant differences observed. However, the analysis revealed a trend suggesting potential associations. Maternal factors were also explored, revealing a significant association between the mother having relatives with OFC and the occurrence of NSOFC in the offspring (*p* < 0.001*). Cases showed a higher prevalence of maternal relatives affected with OFC (27.1%) compared to controls (4.4%). No affected mothers were found in either the cases or the controls. Analysis of parental relationships did not yield statistically significant associations with NSOFC occurrence, whether the father and mother were related, or the nature of their relationship. However, exploration of relationships within family branches, such as 1st cousins, 2nd cousins, and more distant relations, revealed intriguing trends.

**Table 4:** Familial influence on NSOFC: Presence of NSOFC in children by parental OFC status and degree of relationship.

Factors	Cases *n* (%) 88 (46.81)	Control *n* (%) 100 (53.19)	*p* value
Does the father have OFC?			0.014*
Yes	5 (5.88)	0	
No	80 (94.12)	100 (100)	
Does the father have any relatives with OFC?			0.001*
Yes	19 (21.59)	5 (5.32)	
No	69 (78.41)	89 (94.68)	
Does the mother have any relatives with OFC?			< 0.001*
Yes	23 (27.1)	4 (4.4)	
No	62 (72.9)	86 (95.6)	
Are the father and mother related?			0.512
Yes	48 (55.81)	51 (51)	
No	38 (44.19)	49 (49)	
How are the parents related?			0.142
1st cousins (share the same grandparent)	30 (49.18)	31 (50.82)	
2nd cousins (share the same great‐grandparent)	6 (42.86)	8 (57.14)	
More far than 2nd cousins/far relation	11 (73.33)	4 (26.67)	
Non‐related (including the same tribe)	41 (41.84)	57 (58.16)	
Consanguinity			0.787
Consanguineous	36 (48)	39 (52)	
Non‐consanguineous	52 (46.02)	61 (53.98)	

*Note: p* values for differences based on the chi‐squared test for proportions and Fisher's exact test.

* Differences significant at *p* ≤ 0.05.

Moreover, the association between the types of NSOFC and familial history was examined (Table 5), and the contingency table displays the distribution of cases across different categories. No significant association was found between having a familial history of OFC and the occurrence of specific cleft types in the offspring (*p* > 0.05).

**Table 5:** Association between the familial history of OFC and the occurrence of specific cleft types in the offspring.

	CL	CLP	CP	*p* value
History of father with OFC				
Yes	2 (40)	3 (60)	0	0.1603
No	14 (17.5)	44 (55)	22 (27.5)	
Paternal relatives with OFC				
Yes	4 (21.05)	12 (63.16)	3 (15.79)	0.538
No	14 (20.29)	36 (52.17)	19 (27.54)	
Maternal relatives with OFC				
Yes	5 (21.74)	15 (65.22)	3 (13.04)	0.218
No	12 (19.35)	31 (50)	19 (30.65)	

*Note: p* values for differences based on the chi‐squared test for proportions and Fisher's exact test.

Table 6 shows the uOR and aOR and their 95% CIs for the association between various factors with the presence of NSOFC. Children whose fathers had relatives with OFC showed a significantly increased uOR (uOR = 4.90, 95% CI: 0.555–2.623, *p* = 0.002), and after adjusting for potential confounders, the association remained statistically significant (aOR = 8.001, 95% CI: 0.413–3.746, *p* = 0.014). Similarly, the presence of OFC in maternal relatives also showed a significant association with NSOFC, with an aOR of 6.021 (95% CI: 0.430–3.160, *p* < 0.001). Maternal fever during the first trimester was significantly associated with an increased risk of NSOFC (aOR = 3.410, 95% CI: −0.048 to 2.502, *p* = 0.049), whereas bleeding and diabetes during the first trimester were not significantly associated. Conception‐enhancing methods were marginally significant (aOR = 3.051, 95% CI: −0.097 to 2.328, *p* = 0.071). Maternal coffee consumption, other medication use, and diabetes during pregnancy were not significantly associated with NSOFC. The final model was well calibrated (*p* = 0.319, Hosmer and Lemeshow goodness‐of‐fit test), and multicollinearity was not an issue.

**Table 6:** Unadjusted (uORs) and adjusted odds ratios (aORs) and their 95% confidence intervals (CIs) for the association between NSOFC and various factors.

	Unadjusted odds ratio			Adjusted odds ratio		
	uOR	95% CI	*p* value	aOR	95% CI	*p* value
Does the father have any relatives with OFC?						
Yes	4.90	0.555–2.623	0.002*	8.001	0.413–3.746	0.014*
No	Ref			Ref		
Does the mother have any relatives with OFC?						
Yes	7.975	0.965–3.187	< 0.001	6.021	0.430–3.160	< 0.001*
No	Ref			Ref		
Did the mother encounter any of the following during the first trimester?						
None	Ref			Ref		
Fever	8.524	0.617–3.668	0.006*	3.410	−0.048–2.502	0.049*
Bleeding	3.279	−0.485–2.860	0.164	1.153	−1.886–2.172	0.89
Diabetes	0.983	−1.551–1.517	0.983	0.099	−4.608 to −0.179	0.052
Did the mother use any conception‐enhancing methods?						
Yes	3.655	0.229–2.362	0.017	3.051	−0.097–2.328	0.071
No	Ref			Ref		
Did the mother use any other medications during her pregnancy?						
Yes	2.062	0.108–1.339	0.021	1.867	−0.097–2.328	0.119
No	Ref			Ref		
Coffee (number of cups per day)						
None	Ref			Ref		
1 cup/month	0.800	0.244–2.626	0.713	0.935	0.269–1–3.251	0.916
1 cup/day	1.250	0.276–5.653	0.772	0.546	0.096–3.097	0.495
2 cups/day	0.529	0.221–1.271	0.154	0.505	0.197–1.294	0.155
3 cups/day	1.650	0.654–4.165	0.281	1.426	0.537–3.792	0.477

*Note:* Ref, reference category for the variable.

* *p* values reported were significant based on a significance level of 0.05.

For the variable “father's cleft status,” a Firth logistic regression model was applied to account for potential issues related to separation and small sample sizes. This method allows for penalized maximum likelihood estimation, addressing concerns of quasi‐complete separation. The model revealed significant results, providing insights into the familial influence on NSOFC occurrence. The Firth logistic regression model highlights a statistically significant association between the presence of OFCs in fathers and an increased likelihood of NSOFCs in their children. The OR for the presence of cleft in the father was 14.3 (95% CI: 1.47, 176.5) (*p* < 0.001), indicating a 14‐fold increase in the odds of NSOFC in children when fathers have an OFC compared to those without a cleft.

## 4 Discussion

The role of the various risk factors in NSOFC occurrence may vary across different geographical areas or populations. Therefore, this case–control study was designed to investigate the various potential risk factors and identify those associated with NSOFC among Saudi children. MNGHA in Riyadh treats individuals with OFC traveling from different regions across KSA. The study included 188 individuals: 88 individuals with NSOFC and 100 controls (mean age 5.18 ± 2.33 years). The proportion of the different types of OFC in the case group was as follows: cleft lip with or without cleft alveolus (CL) (35.23%), cleft lip and palate (CLP) (39.77%), and CP (25%).

In the present study, birth weight did not show a statistically significant difference between cases and controls (*p* = 0.273), which agrees with several previous studies (Sabbagh et al. 2016; Welch and Hunter 1980; Leite and Koifman 2009). Nonetheless, Bonaiti et al. (1982) conducted a study in France and reported lower birth weight for CPO. Moreover, the age of both parents at delivery showed no significant differences, as the parental age was comparable between the case and control groups. This is in line with several other previous studies (Sabbagh et al. 2016; Krapels et al. 2006; Jamilian et al. 2007; Alsahafi 2010). On the contrary, Herkrath et al. (2012) reported a significant association with parental age in cleft palate‐only cases in a meta‐analysis.

In the present study, no significant association was found between parental socioeconomic status (SES) and having a child with NSOFC (*p* > 0.05). This is in agreement with Sabbagh et al. (2016), although other previous studies suggested that lower maternal or paternal SES and lower education increased the risk of having a child with NSOFC among Iranian populations (Taghavi et al. 2012) and Caucasian Dutch populations (Krapels et al. 2006). This could be explained by the fact that Saudis have relatively higher SES. The mother's residence and marital status during pregnancy in the present study did not show significant differences between cases and controls. Similarly, Sabbagh et al. (2016) found no significant difference between maternal rural and urban living. On the contrary, Alsahafi conducted a study in Jeddah, KSA, and reported that place of residence in rural areas was a significant risk factor for NSOFC (Alsahafi 2010).

The results of the present study showed that paternal smoking did not significantly differ between cases and controls (*p* > 0.05). Similarly, the paternal general smoking status in the study conducted by Sabbagh et al. (2016) showed no significant difference (*p* = 0.36), although there was a higher prevalence of paternal smoking in the cases compared to the controls. However, when they looked at the different types of smoking, paternal water‐pipe smoking was statistically significantly associated with having an infant with NSOFC compared to paternal tobacco smoking. Interestingly, none of the mothers reported smoking in the present study. Smoking among females is quite low and not as common in KSA compared to other countries (Ansari and Farooqi 2017; Health Data 2024). The reported prevalence of tobacco smoking among Saudis was 12.1% (23.7% among males and 1.5% among females). Second‐hand exposure to smoke at home among Saudis was reported to be 17.2% (20.9% for males and 13.1% for females) (Health Data 2024). Moreover, maternal second‐hand smoking had no significant difference in the present study (*p* = 0.672). This is in contrast with the findings reported by Sabbagh et al. (2016), who showed that passive maternal smoking was significantly associated with OFC (*p* = 0.05); they found that 24.5% of the case group reported maternal passive (second‐hand) smoking compared to 15.3% in the control group. Leite and Koifman (2009) reported no significant increase in the risk of OFC for maternal smoking during the first trimester of pregnancy. Although the prevalence of maternal smoking during the first trimester was higher among cases, the OR (1.13, 0.81–1.57) was not statistically significant. They reported that maternal passive smoking during pregnancy was associated with CL/P (1.39, 1.01–1.98), but not with CP. Moreover, they found no association between paternal smoking and OFC occurrence. Taghavi et al. reported that maternal passive smoking increased the risk for CL/P and CP.

Maternal fever during the first trimester was statistically significantly higher in the case group compared to the control group even after the regression analysis (OR = 3.4, *p* = 0.049). Shahrukh Hashmi et al. (2010) showed a significant association between maternal illness and having a child with NSOFC and reported a protective effect with maternal antipyretic medication use. Similarly, a positive relationship between maternal systemic diseases and NSOFC was supported by Taghavi et al. (2012) in a population from Iran. Sabbagh et al. (2016) found a significant association between maternal illnesses and medication use in the 3‐month pregestation period and having a child with NSOFC. However, they did not find a significant association with maternal illness and fever in the first trimester. Maternal use of conception enhancement methods and medications during pregnancy was statistically significant in the present study (*p* = 0.017 and 0.021, respectively). However, the significance disappeared after accounting for confounders using the regression analysis (*p* = 0.071 and 0.119, respectively).

The results of the present study showed no significant effect of consanguinity on NSOFC occurrence. Similarly, Taghavi et al. (2012) reported in a case–control study that there was no significant association between consanguinity and having children with NSOFC in Iran. Alsahafi (Alsahafi 2010) reported that the association between consanguinity and NSOFC was not significant, although they speculated a protective effect. The interesting finding in this study is that having a positive family history of OFC was the strongest predictor, especially from the father's side in the present study (OR = 8, *p* = 0.014). A positive family maternal history had an OR of 6 (*p* < 0.001). Natsume et al. (2000) conducted a case–control study in Japan and reported a higher risk for OFC in children who had a positive family history of OFC (*p* < 0.0001). It was unclear whether syndromic OFC cases were excluded from the study, and no logistic regression analysis was conducted to account for confounders. Leite and Koifman (2009) reported a 14 times higher risk of OFC in children with a positive paternal family history, whereas the risk was 5.96 times higher in children with a positive maternal family history compared to those who had a negative family history of OFC. In KSA, Kumar et al. (1991) and Aljohar et al. (2008) conducted cross‐sectional studies in KSA and found that the prevalence of having a family history of OFC in the cases was 26.8% and 27.8%, respectively. It is important to mention that neither of these studies used a comparison or control group to assess the risk association, and they also did not exclude cases with other associated anomalies. Alsahafi (Alsahafi 2010) conducted a case–control study and found that the case group was almost twice as likely to have a family history of OFC compared to the control group; however, the difference was not statistically significant (OR = 1.9, 95% CI: 0.9–4.3, *p* = 0.1). Sabbagh et al. (2016) reported higher numbers of cases with a family history of congenital abnormalities in the case group (NSOFC) compared with the control group; however, the difference was not statistically significant (*p* = 0.24), and the history taken was not specific for OFC defects only.

### 4.1 Limitations

This study has some limitations that should be considered when interpreting the findings. First, being a retrospective case–control study, it is subject to recall bias, particularly as data were collected through interviews with parents, relying on their memory of past events. Additionally, the study sample, although matched for gender and birth year, was limited to patients from a single healthcare system, potentially affecting the generalizability of the results to the broader Saudi population. Another limitation of the present study is the grouping of all NSOFC types into a single category, which may limit the ability to analyze subtype‐specific risk factors; however, subdividing the data would have affected the statistical power negatively due to sample size reduction. Future research with larger cohorts should explore risk factors for each subtype independently. The exclusion of syndromic cases also narrows the scope of the findings to NSOFCs. Lastly, some variables, such as the use of conception‐enhancing methods, had marginal significance, which may require further investigation with a larger sample size to understand their associations fully.

## 5 Conclusion

The presence of paternal or maternal relatives with OFCs and maternal fever during the first trimester were predictors for NSOFCs, with having affected paternal relatives being the strongest predictor among Saudi children with NSOFCs. Future studies are recommended to verify the present findings and further identify the potential role of gene–environment interaction.

## Author Contributions

All authors contributed significantly to manuscript development. All authors wrote the article and revised it critically for important intellectual content, agreed to submit to the current journal, gave final approval of the version to be published, and agree to be accountable for all aspects of the work. All authors take responsibility for the integrity of the work as a whole from inception to the published article and designated as “guarantor.” The manuscript has been read and approved by all authors, and the requirements for authorship have been met, and each author believes that the manuscript represents honest work.

## Ethics Statement

The Ministry of National Guard Health Affairs Institutional Review Board has reviewed and approved this project (Ref#. RC20/148/R and RSS22R/015/07).

## Conflicts of Interest

The authors declare no conflicts of interest.

## Data Availability

Data are subject to third‐party restrictions.
